# Nanodiamonds Doped with Manganese for Applications in Magnetic Resonance Imaging

**DOI:** 10.1021/acsomega.2c08043

**Published:** 2023-01-22

**Authors:** Srinivasu Kunuku, Bo-Rong Lin, Chien-Hsu Chen, Chun-Hsiang Chang, Tzung-Yuang Chen, Tung-Yuan Hsiao, Hung-Kai Yu, Yu-Jen Chang, Li-Chuan Liao, Fang-Hsin Chen, Robert Bogdanowicz, Huan Niu

**Affiliations:** † Department of Metrology and Optoelectronics, Faculty of Electronics, Telecommunications and Informatics, Gdańsk University of Technology, Gdańsk 80233, Poland; ‡ Accelerator Laboratory, Nuclear Science and Technology Development Center, National Tsing Hua University, Hsinchu 300044, Taiwan; § Department of Biomedical Engineering and Environmental Sciences, National Tsing Hua University, Hsinchu 300044, Taiwan; ∥ Health Physics Division, Nuclear Science and Technology Development Center, National Tsing Hua University, Hsinchu 300044, Taiwan; ⊥ Bioresource Collection and Research Center, Food Industry Research and Development Institute, Hsinchu 300193, Taiwan; # Institute of Nuclear Engineering and Science, National Tsing Hua University, Hsinchu 300044, Taiwan

## Abstract

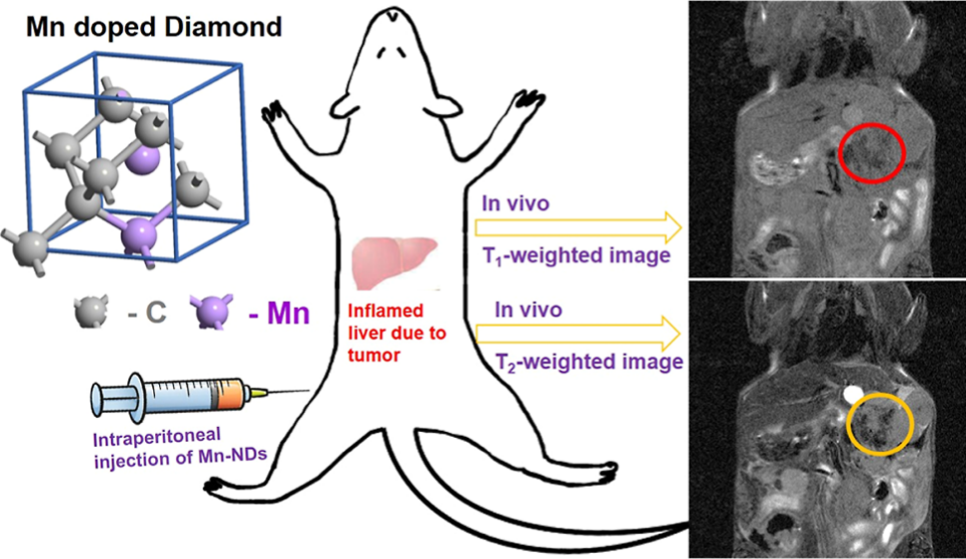

Nanodiamonds (NDs) are emerging with great potential in biomedical applications like biomarking through fluorescence and magnetic resonance imaging (MRI), targeted drug delivery, and cancer therapy. The magnetic and optical properties of NDs could be tuned by selective doping. Therefore, we report multifunctional manganese-incorporated NDs (Mn-NDs) fabricated by Mn ion implantation. The fluorescent properties of Mn-NDs were tuned by inducing the defects by ion implantation and enhancing the residual nitrogen vacancy density achieved by a two-step annealing process. The cytotoxicity of Mn-NDs was investigated using NCTC clone 929 cells, and the results revealed no cytotoxicity effect. Mn-NDs have demonstrated dual mode contrast enhancement for both *T*_1_- and *T*_2_-weighted in vitro MR imaging. Furthermore, Mn-NDs have illustrated a significant increase in longitudinal relaxivity (fivefold) and transversal relaxivity (17-fold) compared to the as-received NDs. Mn-NDs are employed to investigate their ability for in vivo MR imaging by intraperitoneal (ip) injection of Mn-NDs into mice with liver tumors. After 2.5 h of ip injection, the enhancement of contrast in *T*_1_- and *T*_2_-weighted images has been observed via the accumulation of Mn-NDs in liver tumors of mice. Therefore, Mn-NDs have great potential for in vivo imaging by MR imaging in cancer therapy.

## 1 Introduction

Nanodiamonds (NDs) are diamond nanoparticles (size < 100 nm) that exhibit characteristics similar to bulk diamond, such as high hardness, stiffness, chemical inertness, and unique optical and electrical properties.^1^ In addition to bulk diamond properties, the NDs have the advantages of nanomaterials, such as different sizes and large surface areas, which lead to the attachment of various functional groups by surface modification.^2−4^ The surface-modified NDs have been subjected to conjugation with proteins,^5,6^ growth hormones,^7^ fluorescent molecules,^8,9^ and antibodies.^10^ NDs have demonstrated no significant cytotoxicity for different cell lines such as cervical,^11,12^ lung,^13,14^ neuronal,^9^ and renal cells.^15^ The exceptional biocompatibility and ease of functionalization of NDs make them an ideal pathway to attach various drugs and implement them in targeted drug delivery.^16−18^ Therefore, NDs have been utilized in diverse biomedical applications like chemotherapy to cure hepatic cancer stem cells,^19^ gene delivery,^20,21^ tooth root canal treatment,^22^ and antimicrobial agent.^23^

Diamond is a well-known material for its fluorescence properties from the defects caused by doping various elements, resulting in the creation of numerous color centers, which emit light from the UV to IR region.^24^ Nitrogen vacancy (NV) is one of the color centers of diamond, having two charge states NV^o^ and NV^–^. The NV^–^ center is the most prominent emission center due to its unique properties like stable single-photon emission with high quantum efficiency at room temperature, exceptional spin properties, and long coherence times, which allows the NV^–^ to be utilized in quantum computation, quantum information processing, and magnetometry applications.^25−28^ Fluorescent NV^–^-NDs (FNDs) emit bright red luminescence (686–700 nm) with negligible photobleaching, thus increasing the resolution.^29^ FNDs possess a longer fluorescence lifetime than biological tissues, which allows the background-free imaging of FNDS with cells and tissues.^30^ The bright and stable emission of FNDs is paving the way to imagining diamond particles with low cytotoxicity, which enables the monitoring and long-term tracking of single FNDs in living cells.^31^ NDs are functionalized with rare earth chelates for fluorescence imaging and drug delivery applications.^32−34^

In addition, the implementation of NDs displayed great potential for photoacoustic imaging due to the high optical absorbance of NDs.^35,36^ The near-infrared (NIR) fluorescence imaging and photothermal therapy have been performed using NIR probes attached to NDs.^37^ However, the above-mentioned optical-based in vivo imaging methods have illustrated the constraints on tissue penetration, that is, a few centimeters from the surface. To overcome these limitations, NDs have been employed in the magnetic resonance imaging (MRI) technique. MRI is a non-invasive method to obtain exceptional soft tissue contrast, high spatial and temporal resolutions, and deep tissue imaging.^38^ However, the efficacy of the MRI method prominently depends on the contrast agents for the accuracy of diagnosis and enhancement of detection sensitivity.^39,40^ The contrast agents are primarily classified as *T*_1_ contrast [decreases the spin–lattice relaxation time (*T*_1_)] and *T*_2_ contrast [reduces the spin–spin relaxation time (*T*_2_)].^41^ Magnetic carbon dots^42^ and manganese (Mn)-doped nanoparticles^43,44^ have demonstrated their effectiveness as contrast agents in MRI and bioimaging. NDs have been employed as *T*_1_ contrast agents by preparing paramagnetic element [Gd(III)] grafted NDs^45,46^ and paramagnetic element-ND complexes.^47−49^ Paramagnetic elements have shown an influence on reducing the longitudinal relaxation time (*T*_1_). NDs were utilized as *T*_2_ contrast agents by doping ferromagnetic elements into NDs, which decreased the transversal relaxation (*T*_2_).^50,51^

However, some of the contrast agents are visible on both *T*_1_- and *T*_2_-weighted images, which grasp more attention due to functioning as dual-mode contrast agents in MRI.^41,52^ For instance, manganese (Mn^2+^)-doped silica nanoparticles,^53,54^ Mn-doped nanoparticles,^44^ Mn-doped sulfide quantum dots,^55^ and (Mn)–ND conjugates have demonstrated the dual mode contrast enhancement in MRI imaging^47^ due to Mn’s characteristic property of displaying the *T*_1_–*T*_2_ dual-mode contrast ability.^56,57^ However, Mn has an adverse effect on biocompatibility due to its toxicity on cardiotoxicity and neurodegenerative effects.^58,59^ Even though Mn chelates and conjugates were utilized as MRI contrast agents due to their low toxicity and desired relaxivity.^47,60,61^ In addition, according to our best knowledge, very few studies have been reported on Mn-ND-based MRI contrast agents, especially one study that reported ND-Mn dual-mode contrast enhancement for liver tumor detection.^47^ In addition, the Mn chelates or conjugates are prepared by chemical methods and have the probability of leaching during MRI imaging and causing the related toxicity. Therefore, in the present study, Mn-ND conjugates were prepared by a physical method, that is, employing the Mn-ion implantation into ND, to attain the advantage of negligible toxicity due to the tight bonding structure of diamond and hiding from the surface of diamond nanoparticles. Material characterizations were performed to observe the efficacy of Mn doping and the influence of Mn ion implantation on the crystal structure of NDs. The fluorescence properties of Mn-NDs were tuned by a two-step annealing process and systematically investigated by fluorescence microscopy and an in vivo imaging system. The cytotoxicity of Mn-NDs was studied, and then these Mn NDs were subjected as contrast agents for in vitro and in vivo MRI imaging. Mn-NDs demonstrated a significant increase in relaxivities of both *T*_1_- and *T*_2_-imaging of MRI, and enhanced contrast was observed for the in vivo imaging of liver tumors in mice. Therefore, the Mn-NDs showed the ability of dual-mode contrast enhancement in MR imaging.

## 2 Methods

### 2.1 Fabrication of Mn-NDs

ND powder with an average size of 100 nm (Microdiamant Co.) was first dissolved in deionized water (DI water). Then, the ND solution was dispersed onto an oxidized silicon wafer and dried naturally. The ND-coated Si wafers were loaded into the implantation chamber and then implanted by Mn ions with an energy of 80 keV and a dose of 5 × 10^16^ ions/cm^2^. Ion implantation was performed using a 500 kV ion-implanter from High-Voltage Engineering Europe (HVEE, Netherlands). Manganese ions with a charge of +2 extracted from the plasma and selected by an analysis magnet were employed to perform Mn^2+^ ion implantation. The separation process of Mn-implanted NDs from the Si wafer to collect the implanted NDs was described elsewhere.^62^

### 2.2 Material Characterizations of Mn-NDs

Scanning electron microscopy (FESEM, JEOL 6500) was employed to obtain information about the morphology and size of as-received NDs, Mn ion-implanted NDs, MACS filtered Mn-NDs, and filtrate NDs. The bonding characteristics of NDs and Mn-NDs were analyzed by Raman spectroscopy (λ = 532 nm, HORIBA). X-ray photoelectron spectroscopy [(XPS) (PHI Quantera II)] was collected from the NDs and Mn-NDs to attain carbon bonding, phase information, and the efficacy of Mn doping into the diamond. High-resolution transmission electron microscopy [HR-TEM (JEOL JEM-2800F)] was utilized to obtain the microstructure of high-dose Mn ion-implanted NDs and used to calculate the interplanar spacing of crystalline planes.

### 2.3 Fluorescence and Photoluminescence Measurements

In order to obtain the fluorescence and photoluminescence (PL) properties of Mn-NDs, a two-step annealing process was performed on as-implanted Mn-NDs. The two-step annealing process was started with the annealing of as-implanted Mn-NDs at 800 °C for 2 h in a vacuum, and then the vacuum-annealed Mn-NDs were subjected to annealing in the air at 450 °C for 4 h. The annealing process was performed using the Nabertherm annealing furnace. The fluorescence measurements were performed on as-received NDs, as-implanted Mn-NDs, vacuum-annealed Mn-NDs, and two-step-annealed Mn-NDs using a Nikon inverted fluorescence microscope. PL measurements were performed using a micro-PL system (HORIBA, Nd/YAG laser) with a power of ∼100 μW and detector integration time of 1 s. Furthermore, in vivo fluorescence images of the NDs, as-implanted Mn-NDs, vacuum-annealed Mn-NDs, and two-step-annealed Mn-NDs were acquired using the in vivo imaging system (IVIS Lumina II). IVIS fluorescence images were obtained with an excitation light wavelength of ∼550 nm, and the emission was observed around 680 nm.

### 2.4 Cell Viability Tests

The cytotoxicity effect of NDs and Mn-NDs was estimated using the NCTC clone 929 cells in a 3-(4,5-dimethylthiazol-2-yl)-2,5-diphenyltetrazolium bromide (MTT) assay. Mn-NDs and ND samples were sterilized at 121 °C for 30 min. Before adding into cell culture, the Mn-NDs and ND samples were diluted into various concentrations and ultrasonically treated for 30 min. NCTC clone 929 cells were seeded in a 96-well flat-bottom plate as 1 × 10^4^ cell/well and incubated at 37 °C and 5% CO_2_. After overnight culture, Mn-NDs and NDs were added into the medium with designed concentrations of 0.25, 0.125, 0.0625, 0.0313, and 0.0157 mg/mL and incubated for 24 h. In addition, DI water was added as a control. In order to evaluate cell survival, 10 μL of MTT solution was added to each prepared well and incubated for an additional 4 h. Later, the medium was replaced with 100 μL of DMSO and mixed thoroughly to yield soluble formazan. The absorbance was then determined at 540 nm using an ELISA reader and compared to the control solution to measure the cytotoxicity effect, and all conditions were performed in triplicate.

### 2.5 In Vitro MR Imaging and Relaxivity Measurements

Mn-ND solution was prepared by adding it to DI water (five different concentrations ∼ 2.4, 1.2, 0.6, 0.3, and 0.15 mg/mL). DI water and as-received NDs were used as control samples with the same concentrations. Eppendorf tubes were filled with a volume of 0.3 mL of NDs/Mn-ND solution, and a Bruker BIOSPEC 70/30 MRI scanner equipped with proper gradient coils was used for in vitro MR imaging. The DC magnetic field of 7.0 Tesla has been employed to obtain contrast images and relaxation times for *T*_1_- and *T*_2_-weighted images. A multi-slice multi-echo *T*_1_ and *T*_2_ mapping was performed for all the samples. *T*_1_-mapping was obtained from the sixth echo with *T*_R_ = 500 ms and *T*_E_ = 9 ms, and T_2_-mapping was obtained from the 15th echo with *T*_R_ = 2700 ms and *T*_E_ = 484 ms. For both *T*_1_- and *T*_2_-mappings, the matrix size = 256 × 256, the field of view = 60 × 60 mm^2^, and slice thickness = 1 mm have been used. The slope of the inverse of the longitudinal relaxation time (1/*T*_1_ s^–1^) versus concentration plots of Mn-NDs and ND samples was used to attain the relaxivity (*r*_1_) of the MN-NDs and NDs. Similarly, the inverse of the transversal relaxation time of Mn NDs and NDs (1/*T*_2_ s^–1^) was plotted against the concentration, and the relaxivity (*r*_2_) was extracted from the slope of the linear curve.

### 2.6 Preparation of Mn-ND/BSA Conjugates and ND/BSA Conjugates

The BSA attachment to Mn-NDs and NDs was started with a purification and carboxylation process.^63^ The cleaning process was started by subjecting the Mn-NDs and NDs to a mixture of H_2_SO_4_ (9)/HNO_3_ (1) solution at room temperature for 24 h. Subsequently, the purified/oxidized Mn-NDs and NDs were mixed into the 0.1 M NaOH solution at 90 °C for 2 h and then 0.1 M HCl solution at 90 °C for 2 h. After these processes, the solutions were centrifuged to remove the unwanted compounds and achieve carboxylated Mn-NDs and NDs. The retrieved Mn-NDs and NDs were washed several times using DI water to remove the acid and then dried naturally. Then, carboxylated Mn-NDs or NDs with a weight of 10 mg were mixed with 50 mg of bovine serum albumin (BSA) powder (Sigma-Aldrich) in 2 mL PBS and stirred thoroughly. After mixing, the solutions were centrifuged, and the resulting sediment was washed to remove the unattached BSA with Mn-NDS and NDs in order to collect the Mn-ND/BSA conjugates and ND/BSA conjugates.

### 2.7 Fourier Transform Infrared Measurements

In order to confirm the proper attachment of BSA to Mn-NDs and NDs, Fourier transform infrared (FTIR) spectroscopy was used. To perform the FTIR measurements, the carboxylated NDs, carboxylated Mn-NDs, pure BSA, Mn-ND/BSA conjugates, and ND/BSA conjugates were deposited onto the silicon wafers. Then, the absorbance of each sample was measured using a FTIR spectrometer (Bruker IFS66V/S).

### 2.8 Particle Size Distribution and ζ Potential Measurements

To observe the influence of BSA attachment on the dispersion of Mn-NDs, NDs, Mn-ND/BSA conjugates, and ND/BSA conjugates, they were dispersed in PBS with a concentration of 1 mg mL^–1^. The particle size distribution and ζ potential of all samples were measured using a Zetasizer Nano ZS from Malvern Instruments.

### 2.9 In Vivo MR Imaging of Mn-NDs and NDs

The animal model used in this study involved the implantation of tumors in the livers of mice. Briefly, the murine liver tumor BNL 1ME A.7R.1 cell line was purchased from the American Tissue Type Collection and cultured using Dulbecco’s modified Eagle’s medium (Invitrogen) with 10% fetal bovine serum (Invitrogen) and 1% penicillin–streptomycin (Invitrogen) at 37 °C in an incubator containing 5% CO_2_. BALB/c mice (8-week-old males) were intrahepatically inoculated with 20 μL PBS containing 2 × 10^5^ tumor cells. At 15 days post-implantation, mice with liver tumors of similar size were selected for sample injections. Mn-ND/BSA conjugates and control ND/BSA conjugates were prepared in PBS at a 5 mg/mL concentration for intraperitoneal (ip) injection. Coronal *T*_1_-weighted images of mice were obtained using RARE pulse sequences with *T*_R_ = 750 ms, *T*_E_ = 9 ms, matrix size = 256 × 256, field of view = 40 × 40 mm^2^, slice thickness = 0.5 mm, NEX = 4. Coronal *T*_2_-weighted images of mice were acquired using RARE pulse sequences with *T*_R_ = 3100 ms, *T*_E_ = 30 ms, matrix size = 256 × 256, field of view = 40 × 40 mm^2^, slice thickness = 0.5 mm, NEX = 4. Following pre-scanning of the *T*_1_- and *T*_2_-weighted images, solutions of Mn-ND/BSA conjugates or control ND/BSA conjugates (0.25 mL) were, respectively, injected into the mice. At 2.5 h post-injection, coronal *T*_1_ and *T*_2_-weighted images were again acquired to visualize the tumors and thereby surmise the contrast-enhancing effects.

## 3 Results and Discussion

**Figure 1:**
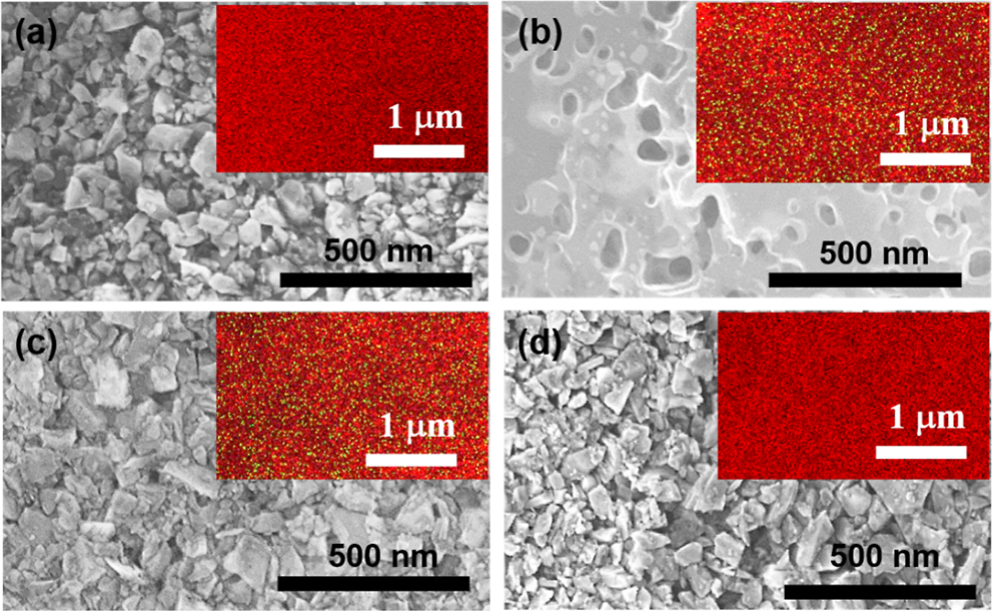
SEM micrographs of (a) NDs deposited on Si wafer prior to Mn ion implantation, (b) Mn ion-implanted NDs, (c) Mn-NDs filtered by the MACS process, (d) filtrate NDs obtained after the MACS filtering process. [The insets are the SEM-EDAS images of the corresponding images; red color represents C, and green color represents Mn].

Figure 1 shows the SEM and SEM-EDAS images that were collected at different stages of preparation of magnetic NDs. Figure 1a displays the SEM image of as-deposited NDs on Si wafer before subjecting it to Mn ion implantation, and these as-received NDs are irregular in shape with an average size of 150 nm. The irregular shape of these NDs originated from the preparation method of the HPHT diamond films. The thickness of the ND layer on Si is not uniform and varies from 3 to 10 μm (a cross-section SEM image has not been shown here). The inset of Figure 1a shows that the SEM-EDAS of the as-received NDs, which describes the presence of only C (red color) of NDs, and the Mn traces were not observed [weight percentage (wt %) of Mn ∼ 0]. SEM images of Mn ion-implanted NDs shown in Figure 1b depicted the ND grain coalescence and resulted in a smooth surface by high-dose Mn ion implantation. The bombardment of metal Mn ions with a high-dose causes lethal damage only on the surface of the ND layer, which causes the trimming of ND facets and results in a smooth surface.^64^ The SEM-EDAS image of Mn ion-implanted NDs shown as the inset of Figure 1b illustrates the presence of Mn ions (green color) with wt % = 0.65 and Mn ions distributed uniformly throughout the NDs matrix. It has to be noted that Mn ion implantation could not assure successful Mn doping to every ND placed on the Si wafer. Therefore, NDs implanted with Mn ions should be separated from non-implanted NDs. For this purpose, the MACS filtering process has been utilized to sort out Mn-NDs; those extracted Mn-NDs and filtrates are shown in Figure 1c,d, respectively. The morphology of extracted Mn-NDs and filtrate NDs is much similar to that of as-received NDs. However, the formation of small diamond particles from breaking large diamond grains by high-dose Mn ion implantation is evidenced. The inset of Figure 1c shows the SEM-EDAS image of the extracted Mn-NDs demonstrating the Mn ions with wt % ∼ 1.65. Whereas the SEM-EDAS image of the filtrate is shown as an inset in Figure 1d, describing the absence of Mn ions with Mn wt % ∼ 0, which confirms the high efficacy of the MACS process for extraction of Mn-NDs.

**Figure 2:**
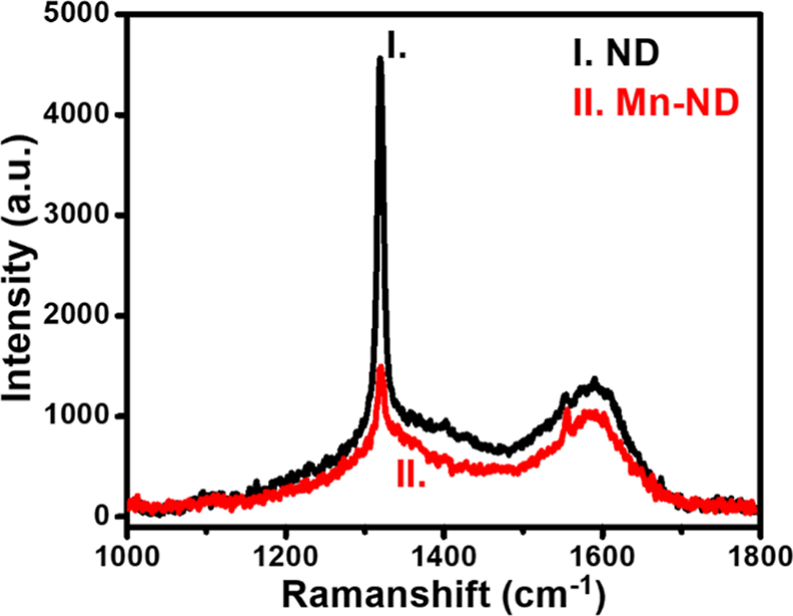
Raman spectroscopy of as-received NDs (I) and Mn ion-implanted NDs (II).

Raman spectra of as-received NDs and Mn-implanted NDs have been collected to observe the influence of high-dose Mn ion implantation on ND bonding structures. Figure 2 shows the Raman spectroscopy results of NDs (curve I) and Mn-NDs (curve II). Raman spectroscopy of as-received NDs displaying the characteristic first-order Raman line of the diamond at 1319.88 cm^–1^ with a full width at half maximum (fwhm) value of ∼ 9.75 cm^–1^ and a broad peak appearing at 1590 cm^–1^ are associated with the presence of sp^2^- carbon (G-band).^65^ Curve II in Figure 2 depicts the Raman spectroscopy of Mn-NDs with the first-order Raman line of the diamond at 1320.75 cm^–1^ (FWHM ∼ 10.51 cm^–1^) and a broad peak at 1590 cm^–1^ (G-band).^65^ The additional sharp peak at the Raman shift of 1554.26 cm^–1^ is from the vibrations of O_2_ molecules for both NDs (curve I) and Mn-NDs (curve II).^66^ However, the Raman peak at 1554.26 cm^–1^ is from the thin layer of SiO_2_ on the Si substrate. The first-order Raman line of natural diamond is centered at 1332 cm^–1^ (FWHM ∼ 5–10 cm^–1^).^67^ However, in this study, NDs and Mn-NDs exhibited the first-order Raman line centered at 1320 cm^–1^. The blue shift of NDs and Mn-NDs is due to the smallness of diamond particles, the stress in the HPHT method-grown diamond films, and phonon confinement.^65,68−70^

**Figure 3:**
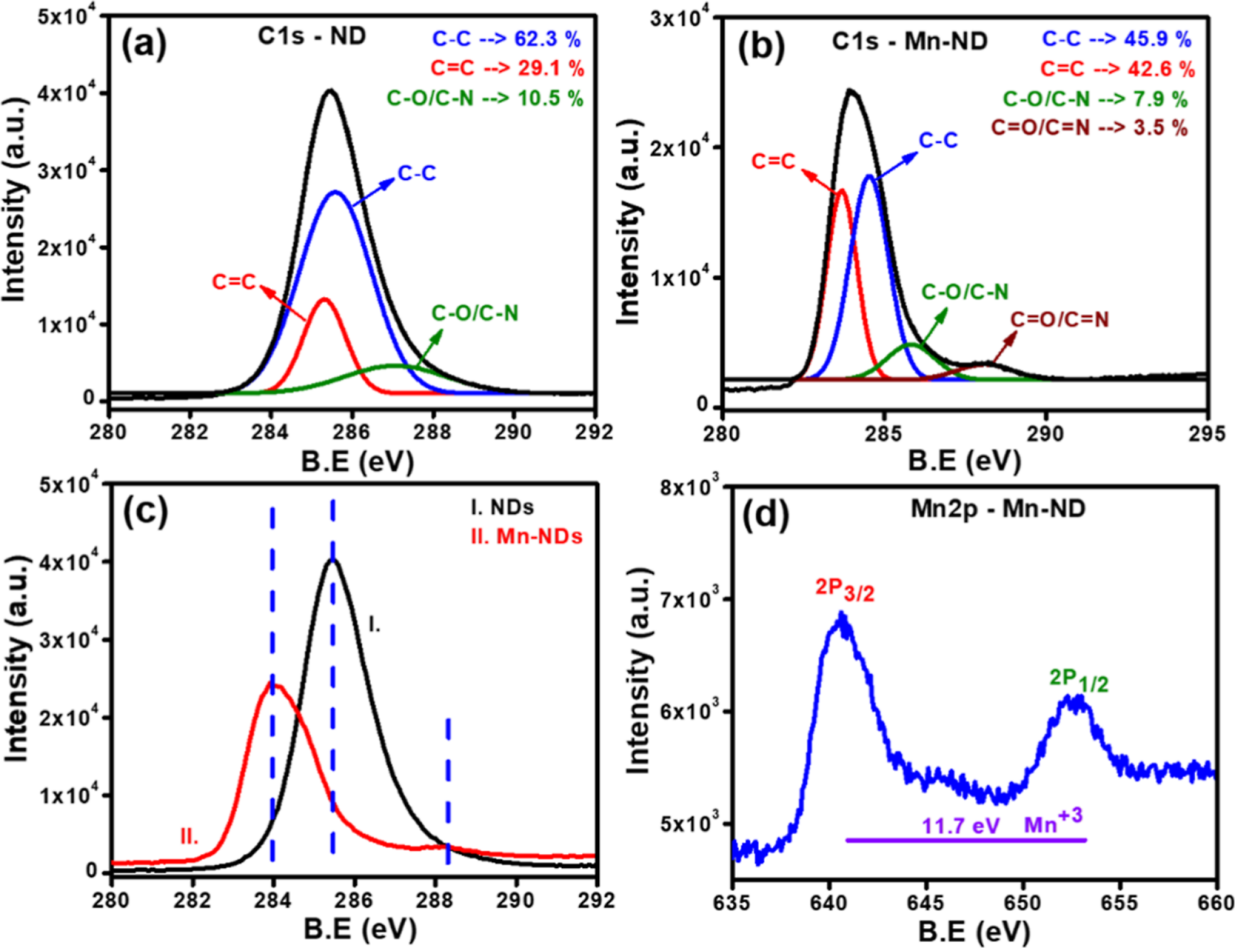
XPS fitted curves of (a) C 1s spectra of as-received NDs, (b) C 1s spectra of Mn-NDs, XPS of (c) C 1s of NDs and Mn-NDs, and (d) Mn 2p spectra of Mn-NDs.

XPS spectra were collected from as-received NDs and Mn-NDs to investigate the impact of high-dose Mn ion implantation on NDs and the efficacy of Mn doping into NDs. Figure 3a illustrates the C 1s core line of as-received NDs; it is further deconvoluted into three Lorentzian peaks such as C–C bonding at 285.6 eV for C-sp^3^ carbon (62.3%), C=C bonding at 285.3 eV for C-sp^2^ carbon (29.1%), and a small peak at 287.1 eV for C–O bonding of O-sp^3^ carbon (10.5%).^65,71−74^Figure 3b illustrates that the spectra of C 1s core line of Mn-NDs have been deconvoluted into four peaks at 284.5 eV for the C–C bonding of C-sp^3^ carbon (45.9%), a peak at 283.7 eV for C=C of C-sp^2^ carbon (42.6%), and other two peaks were observed at 285.8 eV for C–O bonding of O-sp^3^ carbon (7.9%) and a peak at 288.2 eV for O=sp^2^ carbon (3.5%).^65,71−74^ A significant shift to lower energy for the C 1s peak of Mn-NDs was observed due to high-dose Mn ion implantation, which has been displayed by plotting the C 1s core lines of NDs and Mn-NDs in one plot (Figure 3c). The high-dose of Mn ion implantation leads to the amorphization and graphitization on the ND surface. The C 1s peak shift to lower energy after Mn ion implantation is due to surface oxidation and band bending.^75−77^ Furthermore, XPS spectra have been collected to find the presence of Mn ions in the NDs to confirm the successful doping into NDs. Figure 3d illustrates the Mn 2p spectra consisting of the spin–orbit doublet of Mn 2p_3/2_ (640.6 eV) and Mn 2p_1/2_ (652.3 eV) with a binding energy difference of 11.7 eV and the resultant energy difference of the spin–orbit doublet, which reveals that the Mn ions exist as Mn^+3^ in the NDs.^78^ Therefore, the XPS results confirm the significant impact on the diamond surface, the confirmation of Mn doping into NDs, and the Mn charge state in the NDs.

**Figure 4:**
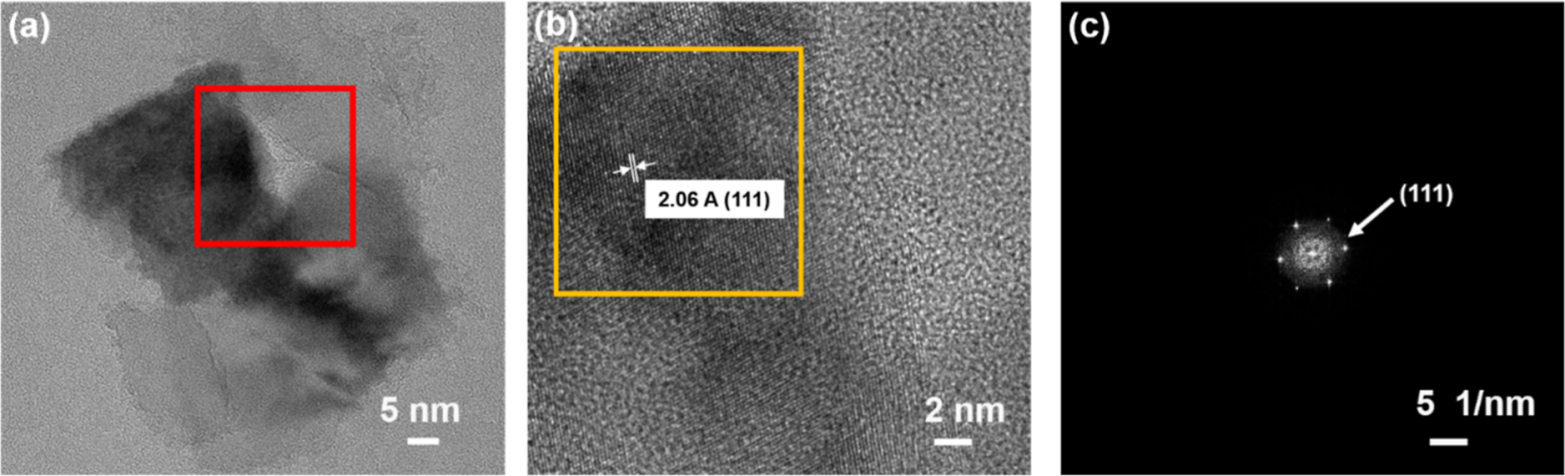
(a) Bright-field TEM image of Mn-implanted NDs, (b) HRTEM image of Mn-NDs from the selected area in (a), and (c) SAED pattern from the selected area in (b).

The influence of high-dose Mn ion implantation into NDs has been investigated using the TEM. Figure 4a displays the bright-field image of Mn-NDs, which divulges irregular-shaped diamond grains of 50–80 nm. A HR-TEM image was collected from the selected area (marked with red color in Figure 4a) to observe the detailed microstructure of Mn-NDs. Figure 4b illustrates the HR-TEM images of Mn-NDs, which describe the diamond’s crystallinity as being preserved even after high-dose Mn ion implantation. However, a non-diamond amorphous carbon exists around the ND grains, resulting from the amorphization by the impact of high-dose Mn ions on ND’s surface.^64,79,80^ The measured interplanar spacing *d* ∼ 2.06 Å reveals the crystalline orientation of the (111) plane of cubic diamond.^81^ Furthermore, a selected area electron diffraction (SAED) pattern has been obtained from the area marked in Figure 4b. Figure 4c depicts the SAED pattern of Mn-NDs disclosing the strong diffraction ring representing the (111) plane of diamond and a diffusive ring at the center due to the presence of amorphous carbon around the Mn-ND particles.

**Figure 5:**
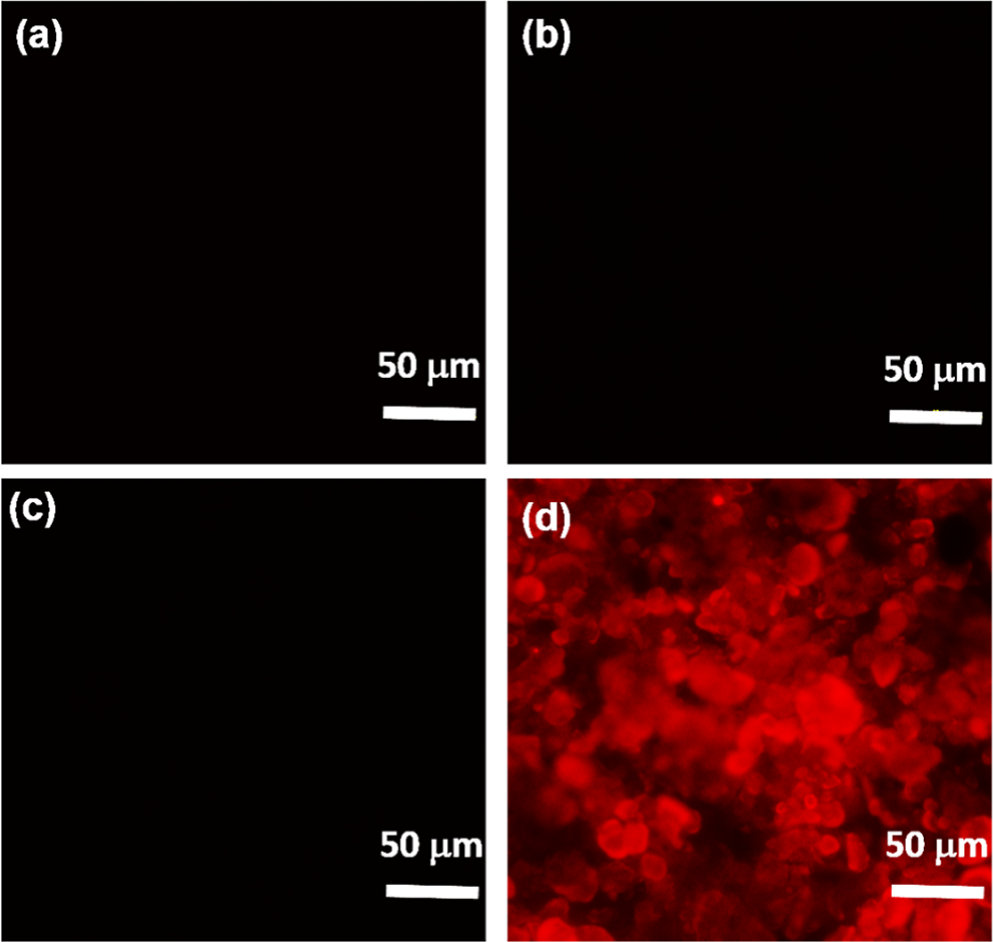
Fluorescence images were observed under a microscope for (a) as-received NDs, (b) as-implanted Mn-NDs, (c) vacuum-annealed Mn-NDs, and (d) two-step-annealed Mn-NDs.

Figure 5a shows the fluorescence image of as-received NDs, which indicates no sign of fluorescence from the NDs because they consist of few NV centers. Even as-implanted Mn-NDs do not show significant fluorescence after Mn ion implantation (Figure 5b). The high dose of Mn ion implantation leads to amorphization on the ND surface, which inhibits the NV’s luminescence. However, the implantation process creates vacancies by dislodging the carbon atoms from lattice sites, and the newly created vacancies require energy to migrate to the vicinity of residual nitrogen atoms to create NV centers.^82^ Therefore, as-implanted Mn-NDs were annealed at 800 °C for 2 h in vacuum to produce NV centers.

Nevertheless, the NV center’s fluorescence has not been observed from the vacuum-annealed Mn-NDs (Figure 5c), which might be due to the quenching of NV fluorescence by the graphitic phase on the surface of Mn-NDs.^83^ The graphitic phase was formed on the surface of Mn-ND by the conversion of amorphous carbon during the annealing process. Therefore, the second step-annealing process was performed at 450 °C in the air to remove the graphitic layer from the surface of Mn-NDs. The graphite phase has been etched out by active oxygen atoms in air annealing, which results in the riddance of the quenching effect on NV emission from Mn-NDs.^84^Figure 5d illustrates the high-intensity red fluorescence from NV centers, which confirms that the two-step annealing process has the potential to prepare highly fluorescent NDs.

**Figure 6:**
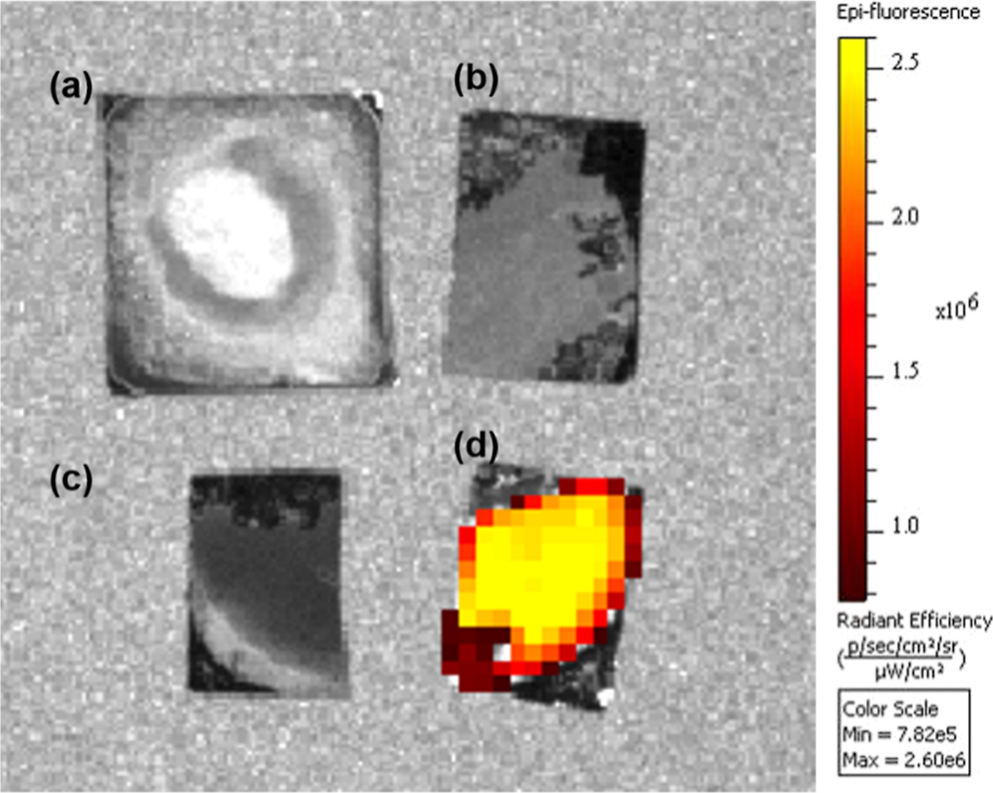
In vivo fluorescence images of (a) as-received NDs, (b) as-implanted Mn-NDs, (c) vacuum-annealed Mn-NDs, and (d) two-step-annealed Mn-NDs.

Furthermore, the fluorescence properties of the NDs and Mn-NDs were observed from the IVIS images. IVIS is a non-invasive and sensitive method to visualize living organisms. However, in the present study, NDs and Mn-NDs were deposited on a Si wafer, and then collected IVIS fluorescence images are shown in Figure 6. Here, fluorescence was observed at a wavelength of 680 nm, and the gray color of these images indicates the absence of luminescence. Figure 6a–c represent the IVIS images of as-received NDS, as-implanted Mn-NDS, and vacuum-annealed Mn-NDs, respectively, which reveals no sign of NV emission, similar to the fluorescence microscopy images (Figure 5a–c). The absence of fluorescence even in the IVIS is also due to few NV centers in NDs, quenching of NV emission by amorphous carbon layer on as-implanted Mn-NDs, and the graphitic layer on vacuum-annealed Mn-NDs. On the other hand, the two-step-annealed Mn-NDs have shown a strong NV emission, which can be seen as a colored area (Figure 6d) that appeared gray in other samples. Therefore, the IVIS and microscope fluorescence measurements confirm that the Mn-NDs consist of a high density of NV centers, but the surface of Mn-NDs should be cleaned to observe the bright red fluorescence.

**Figure 7:**
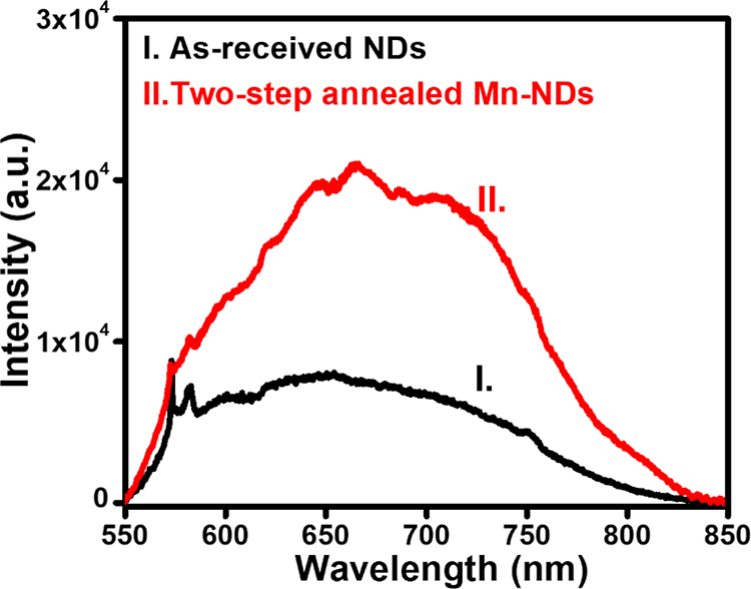
PL spectroscopy of (a) as-received NDs and (b) two-step-annealed Mn-NDs.

Fluorescence microscopy and IVIS images revealed that two-step-annealed samples only illustrated the red luminescence, while other samples did not show any sign of NV luminescence. Therefore, PL measurements were carried out for NDs and two-step-annealed Mn-NDs to cross-check the origin of such fluorescence. Figure 7 depicts the PL spectroscopy of as-received NDs (curve I) and two-step-annealed Mn-NDs (curve II). PL spectroscopy of as-received NDs depicts the typical NV spectrum ranging from 550 to 800 nm,^85^ whereas two-step-annealed Mn-NDs illustrate enhanced NV luminescence.^86^ The enhanced NV luminescence is attributed to three factors: the first one is vacancies created by heavy ions (Mn) implanted in NDs, where Mn ions dislodge the carbon atoms from the lattice sites in the diamond. The second factor is the annealing of Mn-implanted NDs at 800 °C, which leads to the migration of vacancies to the vicinity of residual N atoms in the NDs, resulting in the formation of NV centers. The third one is air annealing of Mn-NDs at 450 °C in the air to remove the thin graphitic layer formed by first-step annealing. It should be noted that two-step-annealed Mn-NDs reveal enhanced NV^–^ emission due to the increased charge conversion of NV^o^ to the NV^–^ charge state of the NV center due to the air oxidation process.^87^ Therefore, the two-step annealing process enhances the PL measurements, confirming the existence of NV centers in as-received NDs; nevertheless, no sign of NV luminescence was observed from fluorescence microscopy images due to the inhibition of NV emission by the presence of a graphite surface.^83^ In addition, emission lines at 573.20 and 582.75 nm are the characteristic diamond Raman lines.^88^

**Figure 8:**
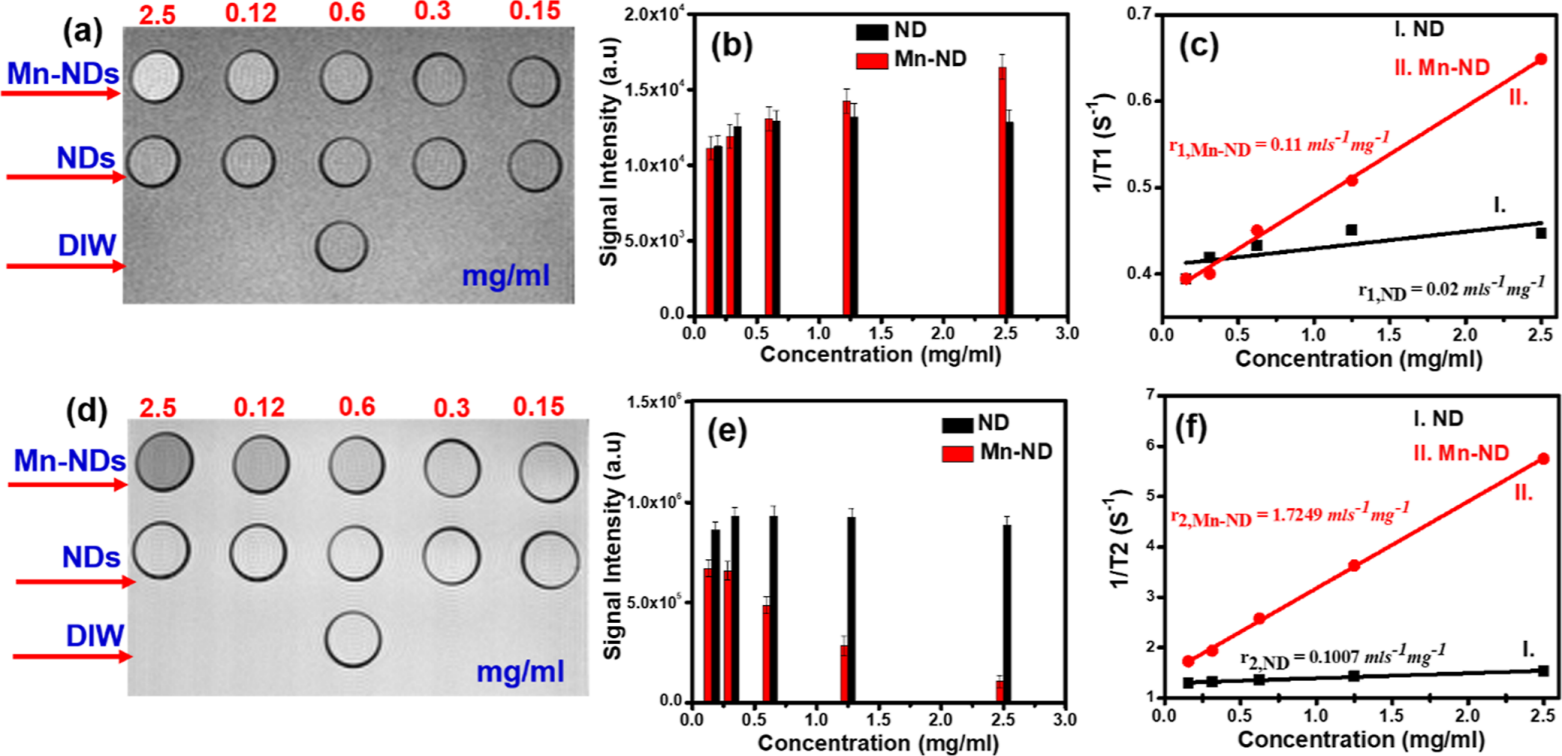
MRI images of NDs and as-implanted Mn-NDs; (a) *T*_1_-weighted contrast images at different concentrations (images collected at 6th echo with *T*_R_ = 500 ms and *T*_E_ = 9 ms), (b) Histogram of intensity vs concentration of *T*_1_-weighted images in (a), (c) inverse of longitudinal relaxation time [1/*T*_1_ (s^–1^)] vs concentration plots, (d) *T*_2_-weighted contrast images at different concentrations (images collected at 15th echo with *T*_R_ = 2700 ms and *T*_E_ = 484 ms) (e) Histogram of intensity vs concentration of *T*_1_-weighted images in (d), and (f) inverse of transversal relaxation time [1/*T*_2_ (s^–1^)] vs concentration plots.

Mn-NDs were demonstrated with the NV center’s fluorescence for cell imaging applications. In addition, the efficacy of Mn-NDs as a contrast agent in MRI imaging was investigated by obtaining the *T*_1_- and *T*_2_-weighted images. Figure 8a shows the *T*_1_-weighted MRI contrast images of Mn-NDs and NDs with varied concentrations from 0.15 to 2.50 mg/mL, which indicates the strong dependence of contrast on the concentration of Mn-NDs. Whereas no significant change in the contrast/brightness was observed for the ND samples for all the concentrations in *T*_1_-weighted images, and these images are similar to the DI water control sample. It is better to represent the comparison of contrast intensity at different concentrations of Mn-NDs and NDs in a histogram (Figure 8b). The resultant contrast intensities of Mn-NDs and NDs were depicted such that the Mn-NDs appear brighter with increasing concentrations, although significant brightness has been observed (compared to control or NDs) at a concentration of 0.60 mg/mL and high brightness is perceived at a concentration of 2.5 mg/mL. Therefore, a positive contrast enhancement was evidenced for Mn-NDs. A prominent decrease in longitudinal relaxation time (*T*_1_) was observed with an increase in the concentration of Mn-NDs, while a slight decrease in *T*_1_ was observed for NDs. The variation in the inverse of *T*_1_ at different concentrations of NDs/Mn-NDs has been plotted to obtain the longitudinal relaxivity (*r*_1_), and the obtained results are shown in Figure 8c. The values of (*r*_1,ND_) and (*r*_1,Mn-ND_) were calculated using the slope of 1/*T*_1_ versus concentration. The value of *r*_1,ND_ ∼ 0.02 mL s^–1^ mg^–1^ was calculated from the slope curve I in Figure 8c, and *r*_1,Mn-ND_ ∼ 0.11 mL s^–1^ mg^–1^ has been calculated using the slope of curve II in Figure 8c. The *r*_1,Mn-ND_ value is five times higher than the *r*_1,ND,_ which confirms that Mn-NDs exhibit a high longitudinal relaxivity compared to the as-received NDs. The higher *T*_1_ relaxation rate is due to the enhanced energy transfer between water protons and Mn ions in NDs by spin–lattice (electron-nuclear) relaxation.

Figure 8d illustrates the *T*_2_-weighted contrast images of NDs and Mn-NDs and NDs for different concentrations, varying from 0.15 to 2.50 mg/mL. The darkness/negative contrast of the Mn-NDs is increased with concentration, while the NDs have not shown any variation in intensity with the increase in the concentration of the ND solution. Figure 8e depicts the histogram of contrast intestines at various concentrations of Mn-NDs and NDs. The contrast intensities of Mn-NDs and NDs have revealed a prominent variation in intensity values for each concentration, and this variation has been increasing with the concentration, that is, Mn-NDs appeared darker at a concentration of 2.5 mg/mL. Therefore, negative contrast enhancement was also demonstrated for Mn-NDs. Furthermore, a sharp and linear decrease in transversal relaxation time (*T*_2_) has been attained with the concentration of Mn-NDs, whereas a small decrease in *T*_2_ was observed for NDs. For the transversal relaxivity (*r*_2_) calculation, the inverse of the *T*_2_ versus concentration has been plotted (Figure 8f). The obtained values of *r*_2,ND_ ∼ 0.1007 mL s^–1^ mg^–1^ (slope curve I in Figure 8f) and *r*_2,Mn-ND_ ∼ 1.7249 mL s^–1^ mg^–1^ (curve II in Figure 8f). The resultant *r*_2,Mn-ND_ value is 16 times higher than the *r*_2,ND,_ demonstrating that Mn-NDs exhibited enhanced transversal relaxivity than the as-received NDs. The enhanced contrast of T_2_-weighted images is due to the local magnetic field inhomogeneity caused by the presence of paramagnetic Mn atoms inside the NDs, which leads to the dephasing of water protons around the Mn-NDs, resulting in extra spin–spin relaxation and a decrease in *T*_2_.

**Figure 9:**
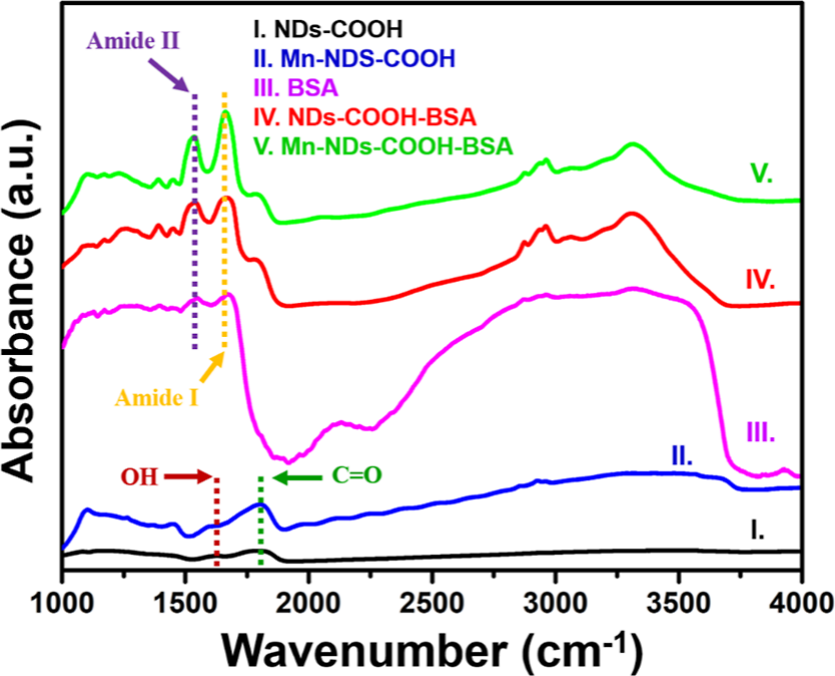
FTIR spectra of carboxylated NDs (I), carboxylated Mn-NDs (II), BSA (III), NDs/BSA conjugates (IV), and Mn-NDs/BSA conjugate (V).

The Mn-NDs/NDs were carboxylated, and subsequently, BSA was attached to reduce aggregation and increase dispersion in the PBS medium. FTIR spectroscopy was employed to investigate the efficacy of carboxylation and BSA attachment to Mn-NDS and NDs. Figure 9 illustrates the FTIR absorbance spectra of carboxylated NDs (I), carboxylated Mn-NDs (II), BSA (III), ND/BSA conjugates (IV), and Mn-ND/BSA conjugates (V). The characteristic peaks of the C=O stretching band of the carboxyl group and O–H bonds were observed for NDs (I) and Mn-NDs (II) as a result of the carboxylation process. Therefore, efficient carboxylation allows for subsequent BSA attachment. Curve III in Figure 9 depicts the FTIR spectra of pure BSA, which indicate the characteristic amide peaks at 1670 cm^–1^ (amide I) and 1535 cm^–1^ (amide II).^89^ The carboxylated Mn-NDs/NDs were attached with BSA as described in the Method section, and the obtained results are shown as curve IV and curve V in Figure 9 for NDs/BSA and Mn-NDs/BSA, respectively. The characteristic amide I and amide II peaks confirm the effectiveness of BSA attachment to NDs/BSA and Mn-NDs/BSA.^89^

**Figure 10:**
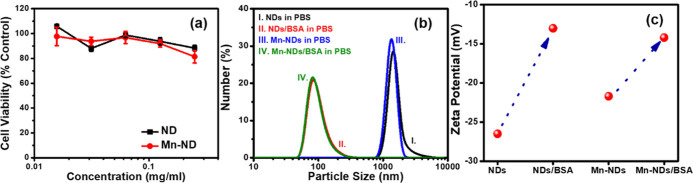
(a) Cytotoxicity test of Mn-NDs and NDs using the NCTC clone 929 cells by the MTT assay; (b) DLS particle size measurements of as-received NDs, Mn-NDs, NDs/BSA conjugates, and Mn-NDs/BSA conjugates; and (c) ζ potential measurements of NDs, Mn-NDs, ND/BSA conjugates, and Mn-ND/BSA conjugates.

The cell viability of NDs and Mn-NDs is crucial for implementing them in cell and animal experiments. Therefore, the cytotoxicity of NDs and Mn-NDs was tested, as described in the methods section, and the obtained results are shown in Figure 10a. The cell viabilities of NDs and Mn-NDs were higher than 80% for the concentrations varying from 0.0157 to 0.25 mg/mL. Thus, the results demonstrate that Mn-NDs do not have significant cytotoxicity. Furthermore, the particle size information of NDs/Mn-NDs is required for in vivo experiments. Thus, particle size measurements were carried out for NDs, Mn-NDs, BSA-attached NDs, and BSA-attached Mn-NDs with a concentration of 1 mg/mL in PBS. Figure 10b displays the DLS particle size measurement results of as-received NDs (curve I), ND/BSA conjugates (curve II), as-implanted Mn-NDs (curve III), and Mn-ND/BSA conjugates (curve IV). The particle size results of NDs and Mn-NDs have demonstrated a particle size higher than 1 μm due to dominant aggregation characteristics of NDs/Mn-NDs due to the presence of graphitic carbon on the surface.

It should be noted that good dispersibility is required, and aggregation of Mn-NDs/NDs should be reduced to employ them for in vivo applications. To achieve such properties, Mn-NDs/NDs were carboxylated and subsequently attached with BSA conjugates. After the BSA attachment to the Mn-NDs/NDs, the dispersibility enhanced significantly, and the aggregation process was suppressed by showing the average size of less than 300 nm for NDs/Mn-NDs in the PBS. Furthermore, zeta potential measurements were performed on the above-described samples in Figure 10b. The ζ potential of Mn-NDs/NDs and Mn-NDs/NDs after carboxylated-BSA attachment is shown in Figure 10c. The resultant ζ potential values revealed an increase in the positive value after BSA attachment for both Mn-NDs and NDs due to the positive ζ values of BSA. It has been reported in previous studies that BSA enhances the stability of NDs in PBS solution and thus improves their dispersibility and averts their aggregation in PBS.^5,90,91^

**Figure 11:**
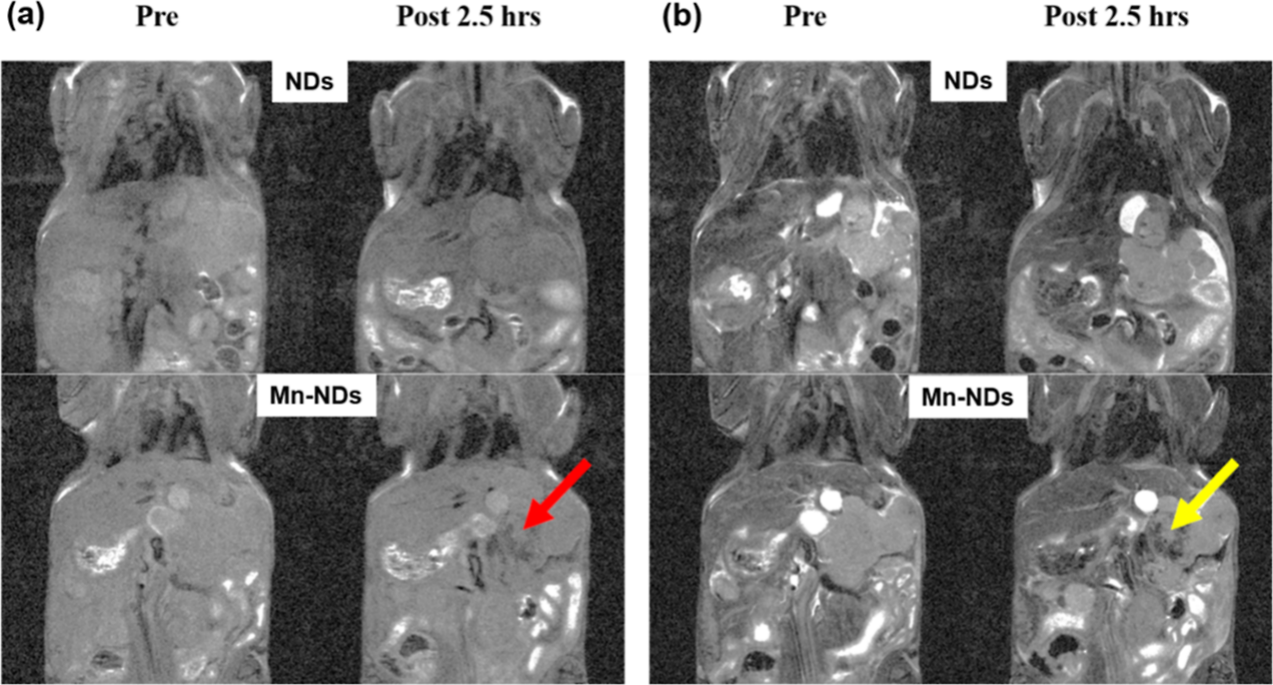
(a) Coronal *T*_1_-weighted images of mice with liver tumors pre and 2.5 h after the injection of Mn-NDs and NDs. The intensity of the liver tumor was measured using ImageJ for all the cases. MR signal intensities of ND (21750) and Mn-ND (20919) for before injection and ND (13176) and Mn-ND (12871) for after 2.5 h of injection were observed from *T*_1_-weighted images of mice with liver tumors. (b) Coronal *T*_2_-weighted images of mice with liver tumors before and 2.5 h after the injection of Mn-NDs and control NDs. MR signal intensities of ND (17358) and Mn-ND (10520) for before injection and ND (14801) and Mn-ND (5820) for after 2.5 h of injection were observed from *T*_1_-weighted images of mice with liver tumors. (The accumulation of Mn-NDs was observed after 2.5 h of injection, while NDs were not detected in *T*_1_- and *T*_2_-weighted in vivo images).

Figure 11 shows the in vivo MR images utilizing NDs/Mn-NDs as contrast agents. Figure 11a depicts the *T*_1_-weighted images of BALB/c mice with liver tumors prior to ip injection and 2.5 h after NDs/Mn-NDs’ ip injection. Before the ip injection of NDs/Mn-NDs, it was difficult for the human eye to identify and distinguish the tumor from normal cells. Even after the ip injection, a very less significant difference has been observed between *T*_1_-weighted images of before and after ip-injected non-magnetic NDs. In contrast, a darkness-intensified area (shown with a red arrow in Figure 11a) has been observed for mice injected with Mn-NDs. The darkness-intensified area is the probable location of the liver tumor. However, a *T*_1_-weighted image should illustrate positive contrast enhancement that is brighter than the surroundings. In the present case, Mn-NDs exhibit intensifying darkness. Figure 11b shows the *T*_2_-weighted images of BALB/c mice with a liver tumor before and 2.5 h after ND/Mn-ND ip injection. Compared with *T*_1_-weighted images, the dark area has been intensified (yellow arrow) in the *T*_1_-weighted image of Mn-NDs, which precisely indicates the location of the liver tumor. However, the NDs do not show the same enhanced contrast in the *T*_2_-weighted image as in the *T*_1_-weighted image.

The role of Mn-NDs as a dual-mode contrast agent in MR imaging depends on the unique properties of NDs as well as the paramagnetic nature of Mn ions. The enhanced contrast for NDs is attributed to the formation of the nanophase of water at the ND-solvent interface due to strong electrostatic potentials on the ND’s facets, leading to surface-mediated attraction on surrounding water molecules.^49,92^ In addition, the paramagnetic Mn ions were placed inside the NDs for the present study; as a result, the Mn-ND material can act as a paramagnetic material. The interaction of paramagnetic Mn-NDs with a surrounding water nanophase leads to enhanced relaxivity.^49^ However, in the present study, the Mn ions are inserted in the diamond core, and the coordination/interaction of paramagnetic ions with water molecules is not a direct process, which might vary with the surface proximity and the concentration of Mn ions of NDs. The enhanced contrast can be attributed to the accumulation of magnetic Mn-NDs in the tumor due to immature blood vessels in the region adjacent to the tumor (enhanced permeability and retention, or EPR effects). Overall, these results demonstrate the potential of Mn-NDs as a dual-mode MRI contrast agent for identifying tumors.

## 4 Conclusions and Perspectives

High-quality Mn-NDs were fabricated by high-dose ion implantation and separated by the MACS filtering process. The efficacy of Mn doping was investigated by SEM-EDAS and XPS methods, and the obtained results indicate the presence of Mn inside the NDs with the Mn^3+^ state. The microstructure information obtained by TEM analysis on Mn-NDs reveals that the crystalline structure has been well preserved even after high-dose Mn implantation. Highly bright NV luminescence was observed from the two-step-annealed Mn-NDs, confirmed by fluorescence microscopy and IVIS imaging. In addition, Mn doping has shown a pronounced impact on enhanced relaxivities for in vitro *T*_1_- and *T*_2_-weighted MR imaging. Furthermore, Mn-NDs have exhibited dual-mode contrast enhancement for in vivo MR imaging of cancer tumors in mice. We are currently working on the influence of higher magnetic fields, ND size, and Mn concentration on the relaxivity of Mn-NDs. Furthermore, we are also investigating the biodistribution of Mn-NDs in mice. In addition, the magnetic property of Mn-NDs has an advantage in attaching cancer drugs and facilitating drug delivery in hyperthermia treatment.
